# Suitability of Goat Colostrum to Produce a Fermented Yogurt-Type Product

**DOI:** 10.3390/ani12213025

**Published:** 2022-11-03

**Authors:** Emilio José González-Navarro, María Carmen Beltrán, María Pilar Molina, Francisco Javier Pérez-Barbería, Ana Molina, María Isabel Berruga

**Affiliations:** 1 Food Quality Research Group, Institute for Regional Development (IDR), Universidad de Castilla-La Mancha, 02071 Albacete, Spain; 2 Institute for Animal Science and Technology, Universitat Politècnica de València, Camino de Vera, s/n, 46022 Valencia, Spain; 3 Biodiversity Research Institute (University of Oviedo-Spanish Research Council-Principado de Asturias), Mieres, 33600 Asturias, Spain

**Keywords:** goat, colostrum, milk, fermented milk, yogurt

## Abstract

**Simple Summary:**

Colostrum is the first milk produced by a mammal after giving birth, and it is awakening a high interest for its use as a sports supplement or nutraceutical due to its higher richness in immunoglobulins, growth factors, hormones, and antimicrobial enzymes than milk. Traditionally consumed by farmers, not much attention has been paid to its potential application in the production of dairy products. As a result of the increased use of artificial lactation in dairy goat systems, a surplus of colostrum is beginning to be found on farms, and the production of fermented milk may be a good opportunity for its use. This work focused on the study of the suitability of goat colostrum to produce a yoghurt-type product and compared its physicochemical, technological, mechanical, microbiological, and sensorial characteristics with those of milk. Results indicate that a colostrum yogurt with a high acceptability by consumers was obtained.

**Abstract:**

The aim of this study was to investigate the potential use of goat colostrum to produce a yogurt-type product as a novel functional dairy food. Four batches of fermented goat colostrum (GCY) were produced using fermented goat milk (GMY) as a reference. Physicochemical, mechanical, and microbial characteristics of cold storage fermented products were evaluated in a weekly basis for 28 days. Sensory analysis was applied to detect potential differences between products and to evaluate the acceptance of GCY by consumers. Results indicate that colostrum showed higher coagulation times than goat milk (480 vs. 350 min to reach pH 4.6). In general, GCY showed a higher protein and fat content and similar features than GMY for most quality parameters, which were highly stable along time. Sensory evaluation led to significant differences between products related to their color and taste. The consumer acceptance test, using a 5 point-Likert scale, showed an overall acceptance of 3.90 ± 0.79 for GCY, with aroma and consistency being the sensory attributes having highest ratings (4.30 ± 0.80 and 4.20 ± 0.96, respectively). Therefore, fermenting goat colostrum with yogurt specific starters could be an interesting alternative to make use of surplus colostrum on farms, allowing for the diversification of commercial goat milk products with potential health benefits for the consumer.

## 1. Introduction

In recent years, colostrum is gaining importance as a potential functional food for humans [1,2,3]. Defined as the first milk produced by a mammal after giving birth [4], its higher richness in immunoglobulins, growth factors, hormones, and antimicrobial enzymes such as lactoferrin, lysozyme or lactoperoxidase than milk makes it an excellent source of bioactive and nutritious compounds [5,6]. For this reason, its use as a sports supplement or as a nutraceutical is gaining interest [1,3,7]; even the approach of using hyperimmune colostrum from vaccinated cows has been suggested as a promising short-term protector for coronavirus infections in human health [8].

Colostrum has been traditionally consumed mainly by farmers in many countries after heating, flavoring, spicing, and/or sugaring [3,4]. However, little attention has been paid to their potential use for making dairy products, probably due to its high content in thermolabile proteins that limits preservation treatments to ensure food safety or to extend the shelf-life [5,9,10]. The use of fermentation with lactic acid bacteria or yeasts seems to be a good alternative to preserve colostrum or even improve its functional properties [1,3].

In recent years, several studies have been conducted focusing on the use of bovine colostrum as a natural ingredient to improve the nutritional value and immune benefits of yogurts, and other fermented milks such as kefir [11,12,13]. However, there is little information on the use of goat colostrum for the production of new dairy products.

In the current goat’s milk production systems in Mediterranean countries [14], the use of artificial lactation is widespread [15,16]. As a result, a surplus of colostrum has been constantly produced with very limited usage, even though the health benefits associated with its consumption are common knowledge. Therefore, the use of goat colostrum to produce new functional dairy foods would be of great interest to the dairy goat sector and an opportunity to diversify the goat milk derivates that are appreciated by consumers given their greater digestibility, lower allergenicity, and beneficial effects on health [17,18]. This study aimed to assess the suitability of goat colostrum in developing a value-added yogurt-type product with organoleptic characteristics acceptable to consumers.

## 2. Materials and Methods

### 2.1. Raw Materials

Colostrum and milk were obtained from a commercial farm of a Murciano-Granadina dairy goat breed that practiced artificial breastfeeding in goat breeding and located close to the dairy pilot-plant of the University of Castilla-La Mancha (Albacete, Spain). Goat colostrum was collected from automatically milking individual animals between the first 24–48 h post-parturition, and immediately frozen in 1-L recipients at −20 °C until use in the laboratory after no more than 15 days had passed. The day before the fermented product elaboration, six colostrum recipients were thawed at 4 °C and mixed in a single sample for each batch. Simultaneously, goat milk was collected from bulk tanks at the commercial farm.

### 2.2. Fermented Products Elaboration

Four independent batches of goat colostrum fermented with yogurt starter cultures, hereinafter “goat colostrum yogurt” (GCY), were produced at a pilot-plant scale. Goat milk yogurts (GMY) were made simultaneously to be used as the reference. Thawed colostrum and unhomogenized fresh milk were filtered and pasteurized at 72 °C for 1 min by using a cooking robot (Thermomix TM 31, Vorwerk, Madrid, Spain). Afterward, they were immediately cooled down at 42 °C, and a commercial culture comprised of a consortium of *Streptococcus thermophilus* and *Lactobacillus delbrueckii* subsp. *bulgaricus* (MY800 LYO 5 DCU, Danisco France, Sassenage, France) was added at a concentration of 0.2 DCU/L. The inoculated dairy matrices were distributed into 100 mL-sterile containers and incubated at 42 °C until a pH of 4.60 ± 0.05 was reached. Then, containers were stored under refrigeration conditions (4 ± 1 °C) until physicochemical, mechanical, and microbial analysis at 7, 14, 21, and 28 days. Changes in the milk and colostrum pH values during fermentation were periodically monitored (15-min) using a pH meter provided by two penetration probes (pH meter HI 5222, Hanna Instruments, Eibar, Spain) and the time, in min, required for reaching the target pH was recorded as the coagulation time.

### 2.3. Physicochemical Analysis

The gross composition (dry matter, fat, protein, and lactose) of the goat milk (GM), goat colostrum (GC), and related fermented products (GMY and GCY, respectively) was analyzed by a mid-infrared spectrophotometer MilkoScan FT6000 (Foss, Hillerød, Denmark). The GC, GMY, and GCY samples were diluted with distilled water (1:1 *w*/*w*) before analysis. The GM and GC samples were also analyzed for somatic cell count by Fossomatic 5000 (Foss). The pH values were checked with a pH meter (GLP 22, Crison, Barcelona, Spain) provided with a penetration probe, and the titratable acidity by the Dornic methodology using 0.111 N NaOH and phenolphthalein as the indicator. According to Novés et al. [19], the fermented products were diluted with distilled water before titration. All parameters were analyzed in duplicate.

### 2.4. Color, Viscosity, and Texture Profile

The CIE L*, a*, and b* coordinates were obtained according to Licón et al. [19] using a Minolta CR-400 colorimeter (Minolta Camera Co., Osaka, Japan) with a CR-a33f cone and a calibrated white plate (Minolta 11333110) with Y = 93.1, x = 0.3160, and y = 0.3323. D65 illuminant and an angle vision of 10° were used.

A rotational viscometer model Visco Basic Plus L (Fungilab, Barcelona, Spain), adapted to a Low Viscosity Adapter LCP/B (Fungilab) was used (spindle rotation from 50 to 100 rpm at 25 ± 0.5 °C) to determine the apparent viscosity in the GM and GC samples. For the fermented products, a rotational viscometer Visco Basic Plus L (Fungilab) fitted with a Heldal unit (Fungilab) was employed, and the measurements were performed with PE and PF spindles with speeds between 0.3 and 1 rpm, and a temperature of 4 ± 1 °C [20].

Texture of the GMY and GCY samples was carried out with a texture analyzer model TA-XT2i (Stable Micro Systems, Surrey, UK) by testing texture “Return to Star” (RTS) in the mode of action of the force compression. A cylindrical aluminum probe (P/10) with a diameter of 10 mm with a 5 kg load cell was used under the following test conditions: crosshead speed of 1 mm/s and penetration distances of 20 mm. Textural parameters (firmness, consistency, and cohesivity) were measured in triplicate.

### 2.5. Microbiological Analysis

Different microbial populations were evaluated in the raw materials and related fermented products. For microbial enumeration, 10 g of the mixed fermented product were homogenized with 90 mL of sterile 0.1% (wt/vol) peptone water (Scharlau, Barcelona, Spain) in a masticator (IUL SA, Barcelona, Spain) for 60 s. Decimal dilutions of the homogenates were prepared with 0.1% (wt/vol) peptone water and seeded in the corresponding medium in duplicate using an Eddy Jet spiral plater (IUL, Barcelona, Spain). Total viable bacteria were enumerated in PCA agar (Scharlau Chemie, Barcelona, Spain) at 30 ± 1 °C for 48 h. For lactic acid bacteria, two culture media were used: M17 agar (Biokar Diagnostics, Barcelona, Spain) with incubation at 37 ± 1 °C for 24 h for *Streptococcus thermophilus* enumeration, and MRS agar, pH 4.6 (Scharlau Chemie) incubated at 37 ± 1 °C for 72 h under anaerobic conditions (AnaeroGenTM, Oxoid Limited, Hampshire, UK) for *Lactobacillus delbrueckii* ssp. *bulgaricus*. Violet Red Bile agar with dextrose was used for enterobacteria incubation (VRBD; Scharlau) at 37 ± 1 °C for 24 h, and molds and yeasts were seeded in Rose Bengal agar (RB, Scharlau) and incubated at 25 ± 1 °C for 120 h. Plates ranging from 30 to 300 CFU were selected for counting in a Countermat Flash (IUL) and microbial counts were expressed as the decimal logarithm of colony-forming units per gram of sample.

### 2.6. Sensorial Analysis of Fermented Products

Sensorial analysis was performed to investigate differences between products perceptible by potential consumers and to evaluate the degree of acceptance of this new dairy product. Semi-trained tasters from the staff members and students at the University of Castilla-La Mancha participated as a sensorial panel.

A total of 87 subjects (40 males and 47 females), ranged 18–67 years old (average age 36.5 years old), participated in a triangle test according to UNE-EN ISO 4120:2004 [21] to evaluate differences between the GMY and GCY products. Sensory evaluation was performed after 14 days of cold storage. Samples were randomly coded with 3-digit numbers, homogenized by gentle stirring, and presented in 50 mL-plastic containers that were stored at 4 °C until testing. The nature of the odd sample and the order of samples in each set were balanced. Each panelist was asked to identify the odd sample, and additionally, to rank the degree of difference of the odd sample, related to the color and taste attributes, in a 3-point scale (1: low difference; 3: high difference). Bread and mineral water were provided to the panelists for mouth-rinsing between samples.

A second set of 47 semi-trained subjects (24 males and 23 females), ranged 20–69 years old (average age 40 years old), conducted a consumer acceptance test for GCY using a 5-point Likert scale (1: disliked extremely; 5: liked extremely). Sensory assessment was conducted after 21 days of cold storage, and tasters were instructed to evaluate the appearance, consistency, aroma, taste, color, aftertaste, acidity, and overall impression.

Acceptability was evaluated as suggested by Gularte et al. [22], who classified a product with good acceptability when the mean values of the sensory properties analyzed were greater than 70% of the maximum value of the hedonistic scale-test employed.

### 2.7. Statistical Analysis

We used non-linear mixed regression models to assess the effect that the type of substrate, milk, or colostrum has on pH along the time required to process the dairy product fermentation. An exponential asymptotic curve to fit the changes in pH at processing time between 50- and 500-min was applied. The time 0–50 min was discarded to make it possible for the pH of milk and colostrum to be represented by the same function. The curves were parameterized using the R software package nlme [23].

The non-linear mixed models evaluated an asymptotic regression function and its gradient of the type,

(1) $$f\left(x\right)=Asym+\left(R0-Asym\right)\times e^{-e^{lrc}\times t}$$

where *t* is processing time in min; *Asym* represents the horizontal asymptote; *R*0 is pH at *t* = 0; and *lrc* is the natural logarithm of the rate constant. The starting values of the parameters of this function were initially estimated with the aid of the self-starting algorithm implemented in the nonlinear least-squares regression package (nlm) in the R software R Core Team [24]. The starting parameters corresponding to the substrates were initially guessed and then optimized by using the new parameters output by the model.

To assess the effects of the type of substrate (milk or colostrum) and storage time (7, 14, 21, and 28 days) on the quality characteristics of fermented products, we used one-way analysis of variance (ANOVA) and the Tukey test at a significance level of *p* < 0.05 to determine differences between means (analyses were carried out using SPSS 24.0 statistical software package, SPSS Inc., Chicago, IL, USA).

## 3. Results and Discussion

### 3.1. Milk and Colostrum Characteristics

Table 1 summarizes the quality characteristics of the raw materials used in this study for the manufacture of fermented products. Colostrum and milk showed similar characteristics to those reported by other authors [25,26] for these dairy matrices in Murciano-Granadina goats, with goat colostrum having a higher titratable acidity and total solids than milk due to its increased content in fat, and especially in protein. Significant differences between products were also detected for color coordinates a* and b*, where goat colostrum resulted more green and yellow than goat milk, which could be related to the higher contents of fat, and protein as well as the riboflavin levels in this matrix [27,28,29]. This higher content in proteins could also be the reason for the significant higher viscosity of goat colostrum, as previously observed in cow matrices [30].

### 3.2. Acidification Kinetics of Goat Colostrum during Fermentation

**Figure 1:**
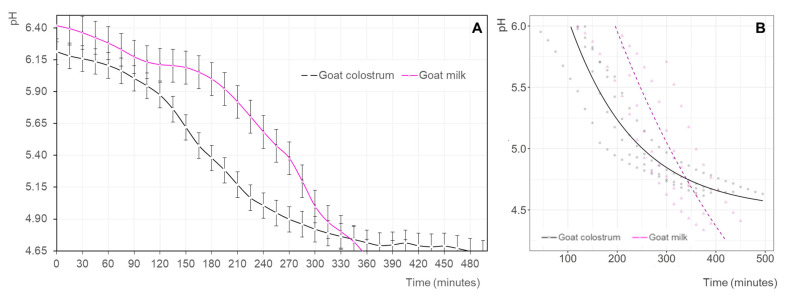
(**A**) Evolution of the pH (mean values ± standard deviation) of the goat milk (pink line) and goat colostrum (black line) inoculated with yogurt starter cultures during fermentation. (**B**) Non-linear mixed regression model of yogurt acidification (pH) predicted for goat milk (dashed pink line; triangle: raw data) and goat colostrum (black line; circles: raw data). The induction period was removed for the fermentation of milk and colostrum in the modeling of the data.

The acidification rate of goat milk (GM) and goat colostrum (GC) during fermentation is presented in Figure 1A. Observations indicate that the two raw materials had different behaviors. Thus, the induction period, in which the pH value remained practically unchanged [31], was shorter for GC, which began to acidify after 90 min of incubation. However, the acidification kinetics after the adaptation period of the lactic acid bacteria to the two substrates was greater for GM, which could be related to the higher buffering capacity of colostrum [29] given its increased protein content. Differences were observed between matrices (*p* < 0.05), reaching the target pH value at approximately 350 and 480 min (Figure 1A), respectively.

A non-linear mixed regression model was applied to predict changes in pH against fermentation time (0 to 500 min). pH values greater than 6.0 were removed to discard the induction period, which allowed us to efficiently use the expected exponential asymptotic curve of changes in pH in both the milk and colostrum products. pH in colostrum decreased steadily up to reaching an asymptotic value of approximately 4.63, whereas in milk, the pH decreased sharply with no indication of reaching an asymptotic value within our fermentation period. The predicted pH of both products equaled at approximately 350 min of fermentation at a value of 4.75 (Figure 1B, Table 2).

### 3.3. Physico-Chemical Characteristics of Fermented Products

Significant differences between the milk and colostrum properties (Table 1) were also detected in the related fermented products. Thus, as expected, higher total solids, fat, and protein contents were found in GCY than in GMY (Table 3) as well as lower lactose content and higher acidity values. These results agree with those reported by other authors when making fermented products from cow milk [12,13,32,33] and buffalo milk [11] fortified with different proportions of bovine colostrum in milk (8–100%). Thus, higher contents of fat, protein, total solids, and lactic acid were obtained in the final products as the proportion of colostrum increased.

The physico-chemical characteristics of GMY and GCY remained stable during cold storage, except for the pH value, having lower average values along time. In both cases, the post acidification that resulted from residual lactose fermentation [34] was relatively low (0.1–0.2 pH units,) which could be related to the commercial cultures used for yogurt production, and changes in the titratable acidity during storage were therefore not significant (*p* > 0.05).

As shown in Table 3, the greatest changes in the pH of the GMY samples took place within the first 14 days of storage (an average decrease of about 0.2 units per week from its storage). However, significant changes for the GCY samples were detected later, between 14 and 21 days of refrigerated storage, which could be related to the higher content of antimicrobial substances in this dairy matrix possibly slowing down the fermentation of lactose, and its higher buffering capacity. In addition, the pH values of GCY were on average higher than those measured in GMY for all of the storage periods considered, which has also been reported by other authors [11,30] making yogurts from milk-colostrum mixtures.

### 3.4. Color and Texture Profile of Fermented Goat Colostrum

Table 4 summarizes the color and texture attributes of the GMY and GCY samples over the cold storage period. In general, similar relative lightness (L*) values were obtained in both types of fermented products for most of the time considered. However, significant differences were detected in the a* (relative redness) and b* (relative yellowness) color coordinates with significantly higher values for b* and inferior for a* coordinate, respectively, in the GCY over the entire storage time. The color differences detected agreed with those observed in the matrices used for yogurt elaborations (Table 1). Similar results were reported by other authors using milk-colostrum mixtures for yogurt production [12,32], where the higher b* values were indicative of an increase as the colostrum percentage was higher; the higher the colostrum, the higher the product yellowness value.

As shown in Table 4, the color attributes of GCY remained stable along time in contrast to those observed in GMY products, showing lower relative yellowness values.

Regarding the mechanical characteristics, the GCY samples had similar viscosity and firmness values, lower consistency, and higher cohesivity than the GMY. In general, the viscosity values of GCY and GMY were similar to those reported by other authors in goat milk yogurts [35]. However, the firmness and consistency values were much lower [36,37]. The lower consistency of goat colostrum yogurt could be related to the lower casein/whey protein ratio in colostrum. It is known that the concentration of whey proteins in colostrum is much higher than that of milk [38], which will undoubtedly affect the structure of the gel obtained after fermentation.

Finally, it should be noted that the viscosity and texture profile of the GCY were subject to less variability than for GMY during storage. In fact, no significant differences were found for GCY, while the firmness, consistency, and cohesivity of GMY were significantly different through cold storage. Other authors [39,40] have also observed a significant decrease in the consistency of yogurts during the first 7–10 days of cold storage in relation to the evolution of the water holding capacity in this period.

### 3.5. Microbial Characteristics of Fermented Products

As shown in Table 5, no significant differences (*p* > 0.05) were detected for microbial populations enumerated in the GMY and GCY samples, which were only affected by the refrigerated storage time. The results herein agree with those reported by Ayar et al. [32], who indicated that colostrum, containing increased concentrations of antimicrobial substances such as immunoglobulins, lactoferrin, lactoperoxidase, lysozyme, and cytokines, does not have an adverse effect on the specific microbial floras of fermented dairy products such as yogurt and kefir.

In general, microbial populations analyzed in the GCY samples remained stable along time (Table 5), except for *Lactobacillus delbrueckii* ssp. *bulgaricus* count, which were significantly higher at the end of the cold storage. Increases in the viable lactobacilli population with time have also been reported by other authors [30] in yogurts from milk containing different proportions of colostrum from bovine, buffalo, and their mixture, which suggest that they could be related to the higher contents of micronutrients in colostrum such as vitamins and minerals, which enhance the growth and activity of yogurt culture [29]. However, a high *Lactobacillus delbrueckii* ssp. *bulgaricus* count was also obtained in the GMY samples at the end of cold storage as well as a lower count for *Streptococcus thermophilus*. No enterobacteria, yeast, or molds were detected in any fermented product along cold storage.

### 3.6. Sensory Evaluation

The discriminant analysis performed using a triangular test found significant differences between the two fermented products for color (*p* < 0.001) and taste (*p* < 0.001) as only one of the 85 panelists was not able to detect the differences between products. When asked about the intensity of these differences, most of the tasters pointed out large differences (Table 6) for both color (42.9%) and taste (46.4%).

**Figure 2:**
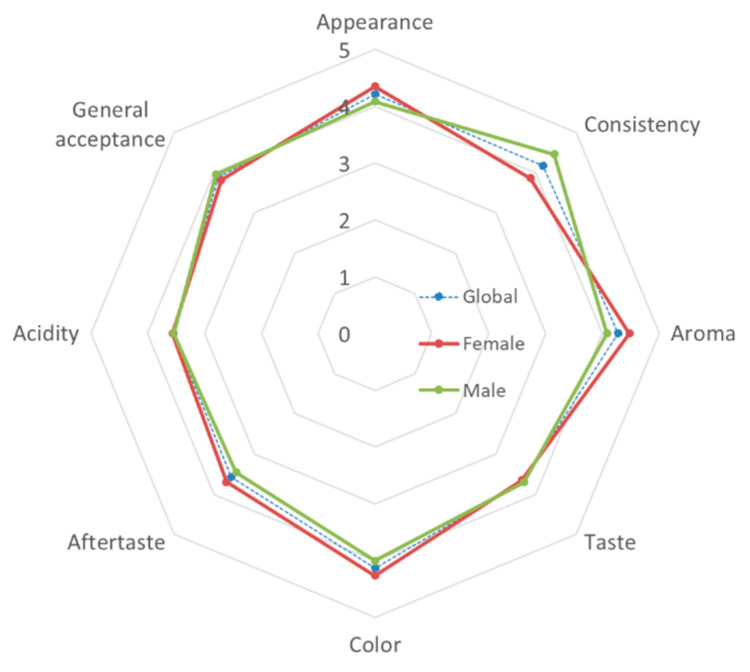
Sensory profile of the goat colostrum yogurt (GCY) using a five-point scale.

Additionally, an affective test using a 5-point hedonic scale was performed to evaluate the consumer acceptance of this new fermented colostrum dairy product. Figure 2 shows the average results obtained, once the data have been transformed to a numerical scale (where 1 = I don’t like it very much, and 5 = I like it very much).

In general, the panelists showed an average overall acceptance close to 4 points (3.89), with acidity being the sensory attribute that received the lowest score (3.55) and aroma the highest (4.27). By gender, men found a significantly more pleasant consistency than women did (*p* = 0.035), while women gave better scores for taste, although the differences did not become statistically significant (*p* = 0.091).

In any case, all of the sensory attributes evaluated in the GCY samples had scores higher than the threshold of acceptability of 70% (3.5 points), which indicate the good acceptability of this fermented product by consumers.

## 4. Conclusions

Fermenting goat colostrum with yogurt specific cultures could be an interesting alternative to take advantage of surplus colostrum in dairy goat farms that practice the ultra-early weaning of kid goats. Results indicate that fermented colostrum has higher protein and fat content, and similar features to those of regular goat’s milk yogurt, although with better aroma and consistency, that remain highly stable throughout the refrigerated storage time. In addition, the acceptability of fermented colostrum by consumers is relatively high, which, together with its potential beneficial effects on health, would allow for diversification of the production of goat milk derivatives by offering a novel functional product that has been appreciated by consumers in recent years.

## Figures and Tables

**Table 1:** Means and standard deviations of some traits of the raw materials used to manufacture yogurt-type fermented products (n = 4).

Parameter	Goat Milk	Goat Colostrum	*p*-Value ^1^
Fat (%)	5.18 ± 0.15	7.80 ± 0.92	0.000
Protein (%)	3.67 ± 0.11	10.62 ± 0.36	0.000
Lactose (%)	4.50 ± 0.12	3.50 ± 0.16	0.000
Solids non-fat (%)	8.98 ± 0.01	14.96 ± 0.43	0.000
Total solids (%)	14.17 ± 0.17	22.64 ± 1.33	0.000
Total viable count (Log cfu/mL)	4.46 ± 0.98	6.93 ± 0.69	0.000
Somatic cell count (Log cel/mL)	6.19 ± 0.25	6.34 ± 0.01	0.276
pH	6.58 ± 0.13	6.36 ± 0.11	0.066
Dornic acidity (ºD)	18.19 ± 2.22	41.40 ± 5.22	0.000
L*	85.01 ± 2.37	84.50 ± 0.30	0.679
a*	−1.91 ± 0.08	−3.67 ± 0.20	0.000
b*	5.59 ± 0.46	9.71 ± 0.20	0.000
Viscosity (cp)	2.66 ± 0.20	7.61 ± 1.30	0.000

^1^*p*-values of ANOVA of differences between milk and colostrum.

**Table 2:** Coefficients and statistics of an exponential asymptotic mixed linear model on pH controlling for product (milk, colostrum). The product reference is milk. Asym represents the horizontal asymptote; R0 is pH at t = 0; lrc is the natural logarithm of the rate constant.

**Random Effects**	**SD**				
R0 (intercept)	0.75				
Residual	0.103				
**Fixed Effects**	**Estimate**	**se**	**df**	**t-Value**	***p*-Value**
Asym (intercept)	4.49	0.047	131	95.12	<0.001
Asym (milk)	−2.22	0.466	131	−4.76	<0.001
R0 (intercept)	7.8	0.442	131	17.67	<0.001
R0 (milk)	0.95	0.149	131	6.42	<0.001
lrc (Intercept)	−4.9	0.081	131	−60.61	<0.001
lrc (milk)	−0.97	0.114	131	−8.5	<0.001

**Table 3:** Mean values ± standard deviation for pH, acidity, and chemical composition of goat milk yogurt (GMY) and goat colostrum yogurt (GCY) (n = 4).

Parameter	Storage Time (Days)	GMY	GCY	*p*-Value ^1^
pH	7	4.39 ± 0.07 ^a^	4.52 ± 0.08 ^a^	0.004
	14	4.19 ± 0.11 ^b^	4.52 ± 0.14 ^a^	0.000
	21	4.12 ± 0.07 ^b^	4.37 ± 0.07 ^b^	0.000
	28	4.19 ± 0.10 ^b^	4.40 ± 0.11 ^b^	0.000
	ANOVA	0.000	0.023	
Acidity (°Dornic)	7	97.08 ± 16.19	127.67 ± 23.71	0.008
	14	91.01 ± 16.95	123.54 ± 15.61	0.001
	21	88.70 ± 6.07	135.08 ± 13.10	0.000
	28	89.11 ± 12.29	134.86 ± 16.09	0.000
	ANOVA	0.595	0.479	
Fat (g·100 g^−1^)	7	6.37 ± 0.20	9.49 ± 2.18	0.001
	14	6.27 ± 0.23	9.00 ± 2.02	0.002
	21	6.59 ± 0.57	8.58 ± 2.01	0.018
	28	6.49 ± 0.42	8.22 ± 2.03	0.035
	ANOVA	0.391	0.163	
Protein (g·100 g^−1^)	7	4.63 ± 0.16	10.12 ± 0.87	0.000
	14	4.74 ± 0.13	9.54 ± 1.08	0.000
	21	4.69 ± 0.18	9.39 ± 1.34	0.000
	28	4.68 ± 0.15	9.89 ± 1.30	0.000
	ANOVA	0.577	0.596	
Lactose (g·100 g^−1^)	7	4.39 ± 0.11	2.90 ± 0.47	0.000
	14	4.37 ± 0.12	2.71 ± 0.46	0.000
	21	4.31 ± 0.14	2.67 ± 0.58	0.000
	28	4.37 ± 0.23	3.06 ± 0.45	0.000
	ANOVA	0.794	0.450	
Dry matter (g·100 g^−1^)	7	15.28 ± 0.35	23.17 ± 2.64	0.000
	14	15.21 ± 0.22	21.86 ± 2.74	0.000
	21	15.43 ± 0.67	19.68 ± 2.62	0.001
	28	15.34 ± 0.47	21.79 ± 3.12	0.000
	ANOVA	0.796	0.122	

^1^*p*-values of ANOVA of differences between GMY and GCY. a,b Different letters within the same column mean significant differences (*p* ≤ 0.05).

**Table 4:** Mean values ± standard deviation for the color (CIE L*a*b*), viscosity, and texture of goat milk yogurt (GMY) and goat colostrum yogurt (GCY) (n = 4).

Parameter	Storage Time (Days)	GMY	GCY	*p*-Value ^1^
L*	7	89.76 ± 1.59	87.44 ± 0.89	0.000
	14	88.03 ± 2.50	87.45 ± 0.83	0.496
	21	87.17 ± 0.91	86.54 ± 1.37	0.291
	28	89.20 ± 3.36	87.19 ± 0.75	0.122
	ANOVA	0.119	0.132	
a*	7	−1.99 ± 0.24	−4.17 ± 0.41	0.000
	14	−1.99 ± 0.54	−4.06 ± 0.40	0.000
	21	−1.77 ± 0.29	−3.44 ± 1.01	0.002
	28	−1.74 ± 0.31	−4.10 ± 0.50	0.000
	ANOVA	0.344	0.067	
b*	7	3.20 ± 1.12 ^a^	10.09 ± 2.11	0.000
	14	1.78 ± 0.44 ^b^	9.23 ± 1.83	0.000
	21	2.06 ± 0.86 ^ab^	7.55 ± 3.66	0.005
	28	3.26 ± 2.14 ^a^	9.23 ± 2.14	0.000
	ANOVA	0.009	0.196	
Viscosity (cp)	7	206,329 ± 79,015	293,076 ± 143,252	0.154
	14	215,753 ± 82,148	249,164 ± 80,424	0.416
	21	217,242 ± 92,147	236,045 ± 55,965	0.575
	28	298,760 ± 74,223	285,709 ± 98,095	0.753
	ANOVA	0.113	0.478	
Firmness (g)	7	30.13 ± 7.01 ^a^	24.13 ± 14.85	0.219
	14	20.13 ± 1.91 ^b^	19.48 ± 3.56	0.614
	21	20.58 ± 2.67 ^b^	22.15 ± 7.81	0.518
	28	21.81 ± 3.40 ^b^	19.89 ± 3.37	0.178
	ANOVA	0.000	0.574	
Consistency	7	435.6 ± 109.7 ^a^	283.3 ± 40.6	0.000
	14	292.2 ± 39.2 ^b^	291.5 ± 46.3	0.767
	21	306.1 ± 37.7 ^b^	281.3 ± 22.9	0.066
	28	312.6 ± 47.1 ^b^	260.1 ± 61.4	0.028
	ANOVA	0.000	0.396	
Cohesivity	7	−17.15 ± 3.28 ^a^	−46.57 ± 95.47	0.000
	14	−11.69 ± 2.04 ^b^	−18.97 ± 3.91	0.000
	21	−11.39 ± 2.09 ^b^	−17.70 ± 3.53	0.000
	28	−11.40 ± 1.90 ^b^	−17.17 ± 7.29	0.015
	ANOVA	0.000	0.397	

^1^*p*-values of ANOVA of differences between GMY and GCY. a,b Different letters within the same column mean significant differences (*p* ≤ 0.05).

**Table 5:** Mean values ± standard deviation for total viable, *Lactobacillus delbrueckii* ssp. *bulgaricus* and *Streptococcus thermophilus* counts in goat milk yogurt (GMY) and goat colostrum yogurt (GCY) during cold storage (n = 4).

Parameter	Storage Time (Days)	GMY	GCY	*p*-Value ^1^
Total viable count (log CFU/g)	7	8.58 ± 0.16 ^ac^	8.49 ± 0.20	0.292
	14	8.14 ± 0.47 ^ab^	8.37 ± 0.38	0.318
	21	7.89 ± 0.18 ^b^	8.84 ± 1.10	0.133
	28	8.89 ± 0.53 ^c^	9.21 ± 0.87	0.440
	ANOVA	0.001	0.096	
*Lactobacillus delbrueckii* ssp. *bulgaricus* count (log CFU/g)	7	8.15 ± 0.39 ^a^	8.32 ± 0.44 ^a^	0.436
	14	8.01 ± 0.49 ^a^	8.29 ± 0.28 ^a^	0.191
	21	8.45 ± 1.19 ^ab^	8.74 ± 1.28 ^ab^	0.726
	28	9.43 ± 0.34 ^b^	9.51 ± 0.34 ^b^	0.662
	ANOVA	0.004	0.003	
*Streptococcus thermophilus* count (log CFU/g)	7	8.72 ± 0.46 ^a^	8.41 ± 0.92	0.167
	14	8.52 ± 0.28 ^a^	8.55 ± 0.17	0.840
	21	8.54 ± 0.25 ^a^	8.65 ± 0.19	0.342
	28	7.96 ± 0.28 ^b^	8.16 ± 0.63	0.577
	ANOVA	0.015	0.061	

^1^*p*-values of ANOVA of differences between GMY and GCY. a,b,c Different letters within the same column mean significant differences (*p* ≤ 0.05).

**Table 6:** Assessment of the color and texture attributes of goat milk yogurt (GMY) and goat colostrum yogurt (FGC) using a triangle test (n = 85 judgments).

Attribute	Differences			
	Slight	Moderate	Large	Inexistent
Color	14	34	36	1
Taste	18	27	39	1

## Data Availability

Not applicable.
